# The differences in drug disposition gene induction by rifampicin and rifabutin are unlikely due to different effects on important pregnane X receptor (*NR1I2*) splice variants

**DOI:** 10.1007/s00210-023-02768-z

**Published:** 2023-10-18

**Authors:** Julie Nilles, Johanna Weiss, Martin Masin, Christopher Tuffs, Moritz J. Strowitzki, Walter E. Haefeli, Stephanie Ruez, Dirk Theile

**Affiliations:** 1 Department of Clinical Pharmacology and Pharmacoepidemiology, Heidelberg University Hospital, Im Neuenheimer Feld 410, 69120 Heidelberg, Germany; 2 Boehringer Ingelheim Pharma GmbH & Co, KG, Birkendorfer Str. 65, 88397 Biberach an der Riss, Germany; 3 Departments of General, Visceral, and Transplantation Surgery, Heidelberg University Hospital, Im Neuenheimer Feld 420, 69120 Heidelberg, Germany

**Keywords:** Pregnane X receptor, Splice variant, Expression, Rifampicin, Rifabutin, Primary human hepatocytes

## Abstract

Rifampicin and rifabutin can activate the pregnane X receptor (PXR, *NR1I2*), thereby inducing pharmacokinetically important genes/proteins and reducing exposure to co-administered drugs. Because induction effects vary considerably between these antibiotics, differences could be due to unequal rifamycin-induced activation or tissue expression of the three major *NR1I2* splice variants, PXR.1 (NM_003889), PXR.2 (NM_022002), and PXR.3 (NM_033013). Consequently, PXR activation (PXR reporter gene assays) and mRNA expression levels of total *NR1I2*, PXR.1, PXR.2, and PXR.3 were investigated by polymerase chain reaction in colon and liver samples from eleven surgical patients, in LS180 cells, and primary human hepatocytes. Compared to the colon, total *NR1I2* mRNA expression was higher in the liver. Both tissues showed similar expression levels of PXR.1 and PXR.3, respectively. PXR.2 was not quantifiable in the colon samples. Rifampicin and rifabutin similarly enhanced PXR.1 and PXR.2 activity when transfected into LS180 cells, while PXR.3 could not be activated. In LS180 cells, rifampicin (10 μM) reduced total *NR1I2* and PXR.3 expression 2-fold after 24 h, while rifabutin (10 μM) increased total *NR1I2*, PXR.1, PXR.2, and PXR.3 mRNA by approx. 50% after 96-h exposure. In primary human hepatocytes, rifampicin (10 μM) suppressed total *NR1I2*, PXR.1, and PXR.3 after 48-h exposure, and rifabutin (10 μM) had no significant impact on total *NR1I2* or any of the splice variants studied. In conclusion, both antibiotics activated the studied PXR splice variants similarly but modified their expression differently. While rifampicin can suppress mRNA of PXR forms, rifabutin rather increases their expression levels.

**Supplementary Information:**

The online version contains supplementary material available at 10.1007/s00210-023-02768-z.

## Introduction

Rifampicin and rifabutin are antibiotics mostly used against mycobacterial infections. Their use is complicated by the high risk of reducing the exposure to co-administered drugs by increasing the intestinal and hepatic expression and activity of proteins being involved in drug metabolism (e.g., cytochrome P-450 isozyme 3A4, CYP3A4) and drug transport (e.g., by P-glycoprotein (P-gp), encoded by *ABCB1*). The risk of drug-drug interactions between rifampicin and rifabutin and other drugs has been known for decades, but the reasons for the considerable differences between the individual rifamycins have not yet been clarified. In general, rifabutin is considered a weaker inducer than rifampicin (Baciewicz et al. 2008; Finch et al. 2002), but detailed comparisons are scarce or misleading because clinical trials always compared the standard doses of 300 mg/day rifabutin with 600 mg/day rifampicin and did not regard the effects of resulting plasma concentrations. Meaningful comparisons of the pharmacodynamics of induction between rifabutin and rifampicin are thus difficult to obtain (e.g., dose-dependent or concentration-dependent CYP3A4 induction). In vitro, we have recently shown that rifabutin poorly enhances *CYP3A4* mRNA or activity when compared to rifampicin, after normalization of effects to intracellular rifamycin concentrations (Nilles et al. 2023). This data thus suggests that molecular differences between the two drugs may explain their unequal inducer properties.

The pregnane X receptor (PXR, encoded by *NR1I2*) is an important nuclear transcription factor that enhances the expression of *CYP3A4* and *ABCB1* upon activation by rifampicin or rifabutin (Chen & Raymond 2006). Besides polymorphisms of the *NR1I2* gene (Svärd et al. 2010; Lamba et al. 2008), transcript variants originating from alternative splicing have been reported. Three major PXR variants have mostly been described (Brewer & Chen 2016): PXR.1 (NM_003889; NP_003880; 434 amino acids) and PXR.2 (NM_022002; NP_071285; 473 amino acids) share exon 2 to 9. The relevant structural difference is that PXR.2 starts from exon 1b (compared to exon 1a in PXR.1), resulting in an additional 39 amino acids at the N-terminus compared to PXR.1. Functionally, these two splice forms are considered comparable (Lin et al. 2009). PXR.3 (NM_033013; NP_148934; 397 amino acids; misleadingly called PXR.2 in Lin et al. 2009) is a truncated protein that harbors an altered ligand-binding domain. Consequently, activation of PXR.3 by rifampicin is weaker than the activation of PXR.1 (“wild-type”). In addition, PXR.3 can counteract the effects of PXR.1 (Lin et al. 2009). Thus far, the variable response of the different splice forms to rifampicin and rifabutin has never been compared as a possible cause of the differences in their drug interaction potential. Finally, there is limited information on the interindividual (Breuker et al. 2014; Liu et al. 2009) variability of mRNA expression levels of *NR1I2* splice variants in LS180 cells, primary hepatocytes, or in major drug-metabolizing human tissues such as the liver.

In consequence, the in vitro activation of PXR.1 to PXR.3 by rifampicin or rifabutin was compared through reporter gene assays. Effects of drug treatment on expression levels in LS180 cells and primary human hepatocytes were assessed. Finally, matched samples of colon and liver specimens were evaluated for mRNA expression levels to record interindividual variability of the splice form expression levels.

## Materials and methods

### Materials

Dulbecco’s modified Eagle’s medium (DMEM) and fetal calf serum (FCS) were purchased from PAN-Biotech (Aidenbach, Germany) and Livetech (Monticello d’Alba, Italy). Phosphate-buffered saline (PBS), medium supplements for LS180 cell culture (glutamine, non-essential amino acids, penicillin/streptomycin), β-mercaptoethanol, dexamethasone, extracellular matrix (ECM) from Engelbreth-Holm-Swarm murine sarcoma, trypan blue solution, and Williams’ medium E were purchased from Sigma-Aldrich (Taufkirchen, Germany). Cryopreserved Hepatocyte Recovery Medium (CHRM) was purchased from Invitrogen (Carlsbad, CA). Phosphate-buffered saline and L-glutamine (GlutaMAX, 100X) were purchased from Thermo Fisher Scientific (Waltham, MA), and insulin-transferrin-selenious acid (ITS), ITS+ (supplemented with bovine serum albumin, 100X), and BD BioCoat collagen I-coated plates were obtained from BD Biosciences (San Jose, CA, USA). Rifampicin and dimethyl sulfoxide (DMSO) were purchased from AppliChem (Darmstadt, Germany) and Sigma-Aldrich (Taufkirchen, Germany). Rifabutin was purchased from Santa Cruz Biotechnology (Heidelberg, Germany) and Cayman Chemicals (Ann Arbor, MI). The Dual-Glo Luciferase Assay System, the pGL4.21 vector, the pGL4.74 [hRluc/TK] *Renilla* vector, the FuGene^®^ HD Transfection reagent, and the CytoTox ONE™ Homogeneous Membrane Integrity Assay were purchased from Promega Corporation (Madison, WI, USA). The *NR1I2* (NM_003889, NM_022002, NM_033013) human cDNA TrueClone^®^ vectors (containing the cDNA of the *NR1I2* splice variant) were obtained from OriGene (Rockville, MD, USA). Qiazol, the RDD buffer, DNAse, the RNeasy Micro Kit (50), and QIAshredder columns were purchased from Qiagen (Hilden, Germany). The High-Capacity Reverse Transcription Kit was obtained from Applied Biosystems (Foster City, CA, USA). Absolute ethanol was purchased from Riedel de Haen (Seelze, Germany). Cell culture flasks and white 96-well plates with white bottom were obtained from Greiner (Frickenhausen, Germany).

### Stock solutions

For experiments with LS180 cells, rifampicin and rifabutin (100 mM stock solutions) were dissolved in DMSO. The stock solutions were diluted with supplemented medium prior to the experiments. The DMSO concentrations in the assays did not exceed 0.1%. For primary human hepatocyte incubations, rifampicin and rifabutin were initially dissolved in ACN/MeOH (1:2) to obtain a 25 mM stock solution. Subsequently, rifamycins were diluted with ACN/MeOH (1:2) to obtain a 5 mM solution. Prior to the treatments, rifamycin solutions were diluted with hepatocyte cell culture medium without supplemented FCS to obtain the final concentration of 10 μM. All stock solutions were freshly prepared prior to the induction experiments. Dexamethasone for hepatocyte cell culture was dissolved in DMSO to obtain a 1 mM solution and was stored at −20 °C.

### LS180 cell culture

LS180 cells originate from a human colon adenocarcinoma and are available at ATCC (Manassas, VA, USA). This cell line shows activity and inducibility of PXR-driven genes or proteins (e.g., CYP3A4 or P-gp) and has consequently been used in many experiments investigating the PXR-mediated induction of drug metabolism or drug transport (Weiss et al. 2013; Harmsen et al. 2008; Gupta et al. 2008; Harper et al. 1991). Moreover, it has been used to assess PXR activation or inhibition through luciferase-based reporter gene assays (Nilles et al. 2022; Weiss et al. 2013). LS180 cells were cultured under standard conditions with DMEM supplemented with 10% FCS, 2 mM glutamine, 100 U/mL penicillin, 100 μg/mL streptomycin sulfate, and 0.1 mM non-essential amino acids.

### Primary human hepatocyte culture

Commercial primary human hepatocytes were purchased from BioIVT (West Sussex, UK; donors: OQA (male), QBU (male), CRT (male)) and Lonza (Basel, Switzerland; donors: HUM183121 (male), HUM190171 (female)). Culturing, quality assessment, and drug treatment were performed as described below. Briefly, cryopreserved hepatocytes were briefly prewarmed and transferred into CHRM thawing medium. Death cells were removed by centrifuging the CHRM medium containing floating cells at 100 g for 10 min at room temperature with braking function set off. Williams’ medium E was supplemented with ITS+ (final concentrations: 6.25 μg/mL insulin, 6.25 μg/mL transferrin, 6.25 ng/mL selenious acid, 5.35 μg/mL linoleic acid, 1.25 mg/mL bovine serum albumin), dexamethasone (final concentration: 0.01 μM), penicillin/streptomycin, and L-glutamine to obtain the primary hepatocyte cell culture medium. A Neubauer chamber was used for cell counting and cell viability was assessed by trypan blue exclusion (only viabilities > 80% were accepted). The cell number was adjusted with hepatocyte plating medium (+ 6% FCS and 0.1 μM dexamethasone) to 550,000 cells/mL (= 55,000 cells/well). Afterwards, cells were seeded on a 96-well collagen I-coated plate and were incubated for 4 h at 37 °C with 5% CO_2_ and 95% humidity. After incubation, an overlay with ECM gel (0.25 mg/mL protein in culture medium with 0.01 μM dexamethasone; 0.1 μM for HUM183121 donor) was performed to generate the final hepatocyte sandwich culture.

### Clinical samples of benign liver and colon tissue

The clinical tissue samples of healthy liver and matched colon had been obtained during the surgical treatment of carcinoma of the rectum or colon, or its metastases. Ethics approval was obtained from the responsible Ethics Committee of the Medical Faculty of Heidelberg University (ethical approval number S-649/2012). All patients gave their written informed consent before being enrolled in the study; their characteristics are listed in Table 1. RNA was isolated as published previously (Shen et al. 2020). Briefly, tissue samples were dispensed with 1 mL Qiazol and stainless-steel beads, and disintegrated using a tissue lyser. The lysate was incubated with 200 μL chloroform for 6 min and centrifuged at 14,800 g for 15 min at 4 °C. Five-hundred microliters of the supernatant was transferred into a new tube, mixed with 500 μL isopropanol, and stored overnight at −20 °C. After centrifugation (14,800 g, 15 min, 4 °C), the supernatant was discarded, and the pellet was washed with ice-cold ethanol (75%), and pelleted again by centrifugation. After drying the sample, the pellet was resuspended with 30 μL distilled water and incubated at 60 °C for 10 min. After adding 3 μL of RDD buffer and 1 μL DNAse, the sample was incubated for 20 min at 37 °C. After centrifugation (14,800 g for 1 min at 4 °C), the RNA contained in the supernatant was transferred into a new tube. Afterwards, RNA concentrations and purity of the samples were determined. The transcriptase-based RevertAid™ H Minus First Strand cDNA Synthesis Kit was used to transfer isolated RNA into cDNA.

**Table 1:** Demographic data of patients the liver and colon samples were obtained from

Patient #	Sex	Age (y)	Neoplastic diagnosis	TNM-classification (8th edition)	Neoadjuvant chemotherapy	Adjuvant chemotherapy	Comorbidity
1	Female	55	Rectum carcinoma	pT3pN1M0	No	Yes	Hypothyroidism, bronchial asthma
2	Female	56	Metastasized rectum carcinoma	pT3pN1M1	Yes	Yes	Hypertension
3	Male	58	Metastasized rectum carcinoma	pT2pN1M1	No	Yes	Hypertension, cervix carcinoma
4	Female	73	Metastasized colon carcinoma	pT3pN2M1	No	No	Chronic obstructive pulmonary disease
5	Male	70	Metastasized rectum carcinoma	pT4pN2M1	No	Yes	Acoustic neuroma
6	Male	87	Metastasized rectum carcinoma	pT3pN2M1	No	Yes	Hypertension
7	Female	64	Metastasized colon carcinoma	pT2pN1M1	Yes	Yes	Hypertension
8	Female	55	Metastasized rectum carcinoma	pT2pN2M1	No	Yes	Epilepsy
9	Female	76	Metastasized colon carcinoma	pT4pN2M1	No	Yes	Type 2 diabetes mellitus
10	Male	58	Metastasized caecum carcinoma	pT1pN1M1	Yes	Yes	Hypertension, coronary heart disease, non-alcoholic fatty liver disease, obesity
11	Male	60	Metastasized colon carcinoma	pT3pN0M1	Yes	Yes	Hypertension, coronary heart disease

### Growth inhibition assay

Because growth-inhibiting effects can confound the results obtained in cell culture, potential antiproliferative effects of rifamycins were investigated in LS180 cells prior to incubation experiments by crystal violet staining as published previously (Peters et al. 2006). Briefly, seeded cells were exposed to different rifamycin concentrations for 24 h, 96 h, or 144 h. After incubation, cells were washed with PBS and subsequently stained with 50 μL crystal violet for 15 min at room temperature on a rotary shaker. Afterwards, cells were washed thrice with double distilled water to remove unbound dye. Experiments were performed in three independent biological replicates with quadruplets for each concentration. Absorption of crystal violet (555 nm) was recorded after dissolving the wells with 200 μL methanol (SpectraMax iD3 from Molecular Devices, Wokingham, UK), and mean background values were subtracted from the measured absorbance values of the cell samples. The proliferation of untreated cells was set to 100%. Only rifamycin concentrations below the IC_20_ value were used in all subsequent experiments.

### Lactate dehydrogenase assay

Possible cytotoxic effects of the rifamycins on primary human hepatocytes were evaluated with the CytoTox ONE™ Homogeneous Membrane Integrity Assay (Promega Corporation, Madison, WI, USA) according to the manufacturer’s instructions. This fluorescence-based system detects lactate dehydrogenase activity (LDH) in the supernatant when the cell membrane is disrupted. Briefly, 50 μL of cell culture supernatant was transferred after the treatment period to a black 96-well plate, and 50 μL of CytoTox ONE™ reagent (including LDH substrate) was added. After brief shaking, plates were incubated for 7 min at room temperature under light protection. Fluorescence was recorded with a Tecan Spark plate reader (excitation: 545 nm, emission: 590 nm) after adding 25 μL stop solution. A lysis control, provided by the manufacturer, served as a positive control. To determine cytotoxic effects, the mean background values of the background fluorescence were subtracted from the measured fluorescence values of the samples. Untreated cells were set to 100%. Cytotoxic effects were assessed for each sample/well.

### PXR activity assay

The methodology of the PXR reporter gene assay has been described previously (Weiss et al. 2013; Nilles et al. 2022). Seeded cells (50,000 LS180 cells per well) were transfected with 20 ng of the respective PXR splice variant expression vector, 80 ng of the reporter vector (PXR response elements of the CYP3A4 gene, cloned upstream of the firefly luciferase open reading frame), and 10 ng of the *Renilla* vector (pGL4.74 [hRluc/TK]), used for signal normalization. Twenty-four hours after transfection, cells were treated with rifampicin or rifabutin for 72 h, and the resulting luminescence (firefly and *Renilla*) was recorded with the SpectraMax iD3 luminometer, using the Dual-Glo Luciferase assay system according to the manufacturer’s instructions. Experiments were performed in three independent biological replicates with quadruplets for each concentration. Because preliminary control experiments had demonstrated variable antiproliferative effects of PXR transfection (PXR.1 through PXR.3) and drugs (rifampicin, rifabutin), the distinct exposure concentrations were adapted accordingly to prevent toxic adverse effects and thus differ between the experiments.

### Sample preparation of LS180 cells

LS180 cells were seeded (500,000 cells/flask) and allowed to grow for 3 days. Afterwards, cells were induced with 10 μM rifampicin or rifabutin for 24, 96, and 144 h. After each induction period, total RNA was isolated using the GenElute™ Mammalian Total RNA Miniprep Kit according to the manufacturer’s instructions. The transcriptase-based RevertAid™ H Minus First Strand cDNA Synthesis Kit was used to transfer isolated total RNA into cDNA. Experiments were performed in four independent biological replicates.

### Sample preparation of primary human hepatocytes

One day after the ECM overlay, primary human hepatocytes were exposed to 10 μM rifampicin or rifabutin for 48 h. After 24 h of incubation, the induction medium was replaced with freshly prepared rifamycin-containing medium. After the entire induction period (48 h), possible cytotoxic effects were assessed with the LDH cytotoxicity assay. Then, total RNA was isolated using the RNeasy Micro Kit according to the manufacturer’s instructions with minor changes. Briefly, cells were lysed with 60 μL RLT buffer (provided by the manufacturer) containing 10 μL/mL β-mercaptoethanol. Three independent replicates were pooled to obtain one final sample with a sufficient amount of total RNA (total lysis volume: 180 μL/replicate). Then, the lysate was transferred to a QIAshredder column and was centrifuged for 2 min at 14,000 rpm to gain optimal RNA extraction. The isolation procedure also included the optional DNase I incubation. Total RNA was eluted with 14 μL RNAse-free water. Subsequently, 150 ng total RNA was applied to synthesize the cDNA using the High-Capacity Reverse Transcription Kit with a final volume of 20 μL. Twenty microliters of RNAse-free water was then added and cDNA was stored at −20 °C.

### Evaluation of PXR splice variant expression levels

**Fig. 1:**
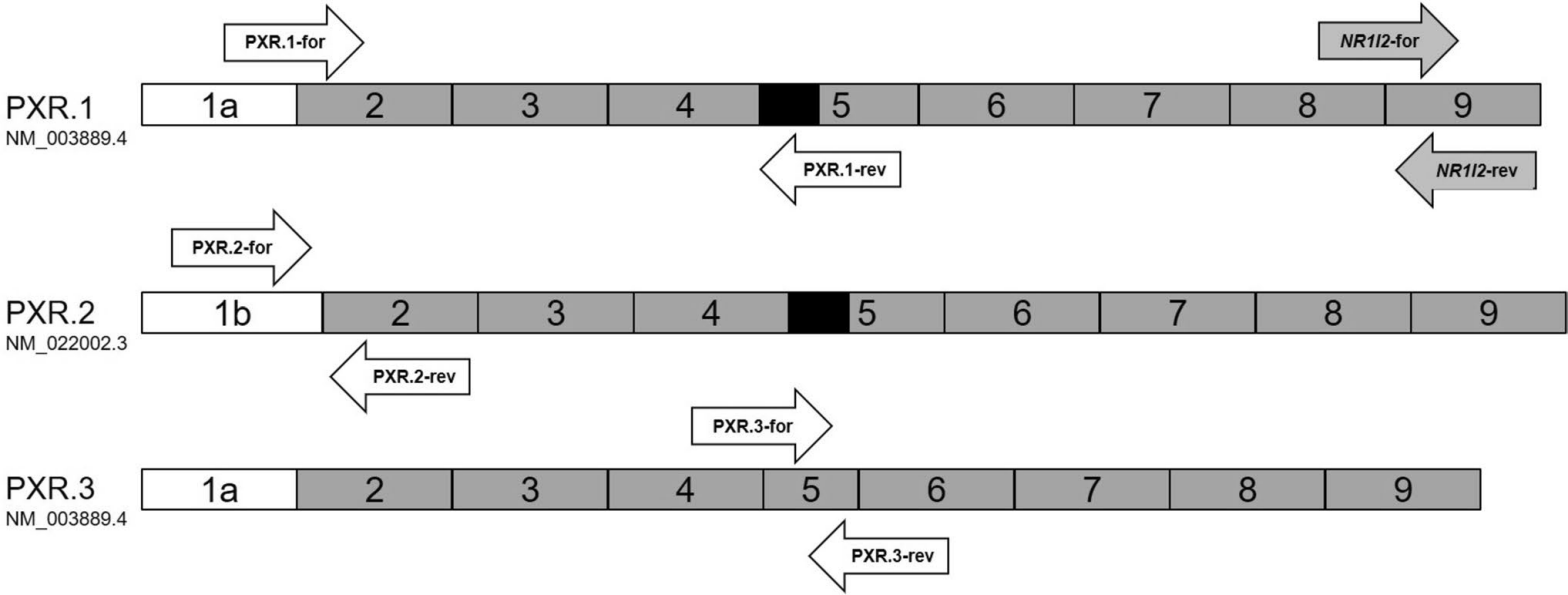
Structure of the three *NR1I2* mRNA transcripts (PXR.1, PXR.2, PXR.3) and binding sites of the respective primer pairs. Exons 2 through 9 are represented as gray rectangles, the alternative exon 1 (1a or 1b) is shown in white. The region of exon 5 that is only expressed in PXR.1 and PXR.2 is highlighted in black. The primer pair for total *NR1I2* (*NR1I2*-for; *NR1I2*-rev; gray arrows) binds to exon 8/9 (forward primer) and 9 (reverse primer). Primer pair combinations only for *NR1I2* splice variants bind to regions being selectively present in the respective transcript variant. PXR.1-for recognizes PXR.1 and PXR.3; PXR.1-rev recognizes PXR.1 and PXR.2; PXR2-for recognizes only PXR.2; PXR.2-rev recognizes PXR.1-3; PXR.3-for recognizes only PXR.3; PXR.3-rev recognizes PXR1-3. The combination of PXR.1-for/rev, PXR.2-for/rev, and PXR.3-for/rev is thus selective for only one splice variant

*NR1I2* splice variant expression levels were quantified by real-time reverse transcription (RT) polymerase chain reaction (PCR) with the LightCycler^®^ 480 (Roche Applied Science, Mannheim, Germany). Primers (sequence, nucleotide position) used are listed in Table 2 and the approximate location of the primers is depicted in Fig. 1. In LS180 cells, hypoxanthine-guanine phosphoribosyltransferase was the most stable housekeeping gene under the experimental conditions (tested with geNorm, version 3.4, Center for Medical Genetics, Ghent, Belgium) and was thus used for normalization. For human colon and liver tissue samples and the primary hepatocytes, the human acidic ribosomal protein and the ribosomal protein L13 were used for normalization. Data were analyzed as described previously (Albermann et al. 2005). Analysis of primary hepatocytes (untreated and treated) and tissue samples was performed in two technical replicates. LS180 cell analysis was performed in four independent experiments for each treatment and analyzed with two technical replicates.

**Table 2:** Sequences and nucleotide positions of primer pairs for the detection of total *NR1I2* and the three *NR1I2* splice variants PXR.1, PXR.2, and PXR.3

Name	Primer sequence	Nucleotide position^#^	Reference
PXR.1-forward	5′-ACT TAC CAC CAA GCA GTC C-3′	185 to 203	*
PXR.1-reverse	5′-TCA AAG AGC ACA GAT CTT TCC-3′	830 to 850	*
PXR.2-forward	5′-AAG GAC AGC AGC ATG ACA G-3′	37 to 55	*
PXR.2-reverse	5′-CAA AGT CAG CAT GGT TCC AG-3′	189 to 208	*
PXR.3-forward	5′-TCA AGA ATT TCC GGG TCT CTC-3′	728 to 748	Lamba et al. (2004)
PXR.3-reverse	5′-CGA TGG GCA AGT CCC TGA AG-3′	900 to 919	Lamba et al. (2004)
Total *NR1I2*-forward	5′- CCC AGC CTG CTC ATA GGT TC- 3′	1367 to 1386	Svecova et al. (2008)
Total *NR1I2*-reverse	5′-CTG TGA TGC CGA ACA ACT CC-3′	1500 to 1519	Svecova et al. (2008)

^#^Primer positions were evaluated by the online tool available at https://www.bioinformatics.org/sms2/primer_map.html using the respective cDNA sequences available at https://blast.ncbi.nlm.nih.gov/Blast.cgi?PROGRAM=blastn&PAGE_TYPE=BlastSearch&LINK_LOC=blasthome

*Primers were designed for this study by means of the online tool available at https://eurofinsgenomics.eu/de/ecom/tools/pcr-primer-design/

### Statistics

Statistical analysis was performed using GraphPad Prism version 9.1 (GraphPad Software Inc., La Jolla, CA, USA). Differences in the pharmacodynamics (EC_50_, *E*_max_ of reporter gene assay) of rifampicin and rifabutin were evaluated by non-parametric Mann-Whitney test. The impact of drug treatment on *NR1I2* or its splice variant expression in primary hepatocytes or LS180 cells was evaluated by non-parametric Kruskal-Wallis test and Dunn’s test, correcting for multiple comparisons. Differences of *NR1I2* or its splice variant expression in colon vs. liver samples were evaluated by non-parametric Wilcoxon matched-pairs signed-rank test. Outliers in the clinical sample data set were identified by ROUT testing at a Q-level of 1% and were subsequently excluded from analysis.

## Results

### Rifamycin-mediated activation of *NR1I2* splice variants

**Fig. 2:**
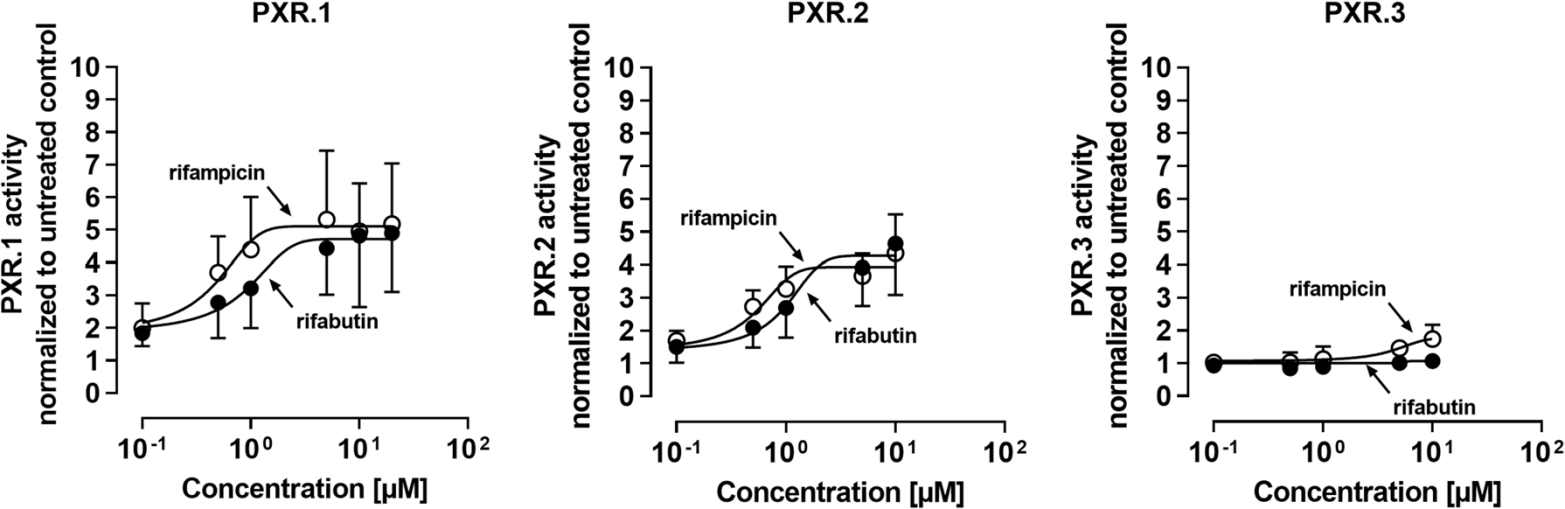
Relative PXR reporter activity in LS180 cells after selective overexpression of *NR1I2* splice variants (PXR.1, PXR.2, or PXR.3) and subsequent 72-h exposure to rifampicin (open circles) or rifabutin (closed circles). Data shown is the mean ± S.D. of three independent biological replicates with triplicates for each concentration. Data were fitted according to an *E*_max_ model (4-parameter logistic equation; variable with slope)

In LS180 cells transfected with the PXR.1 overexpression vector, both rifampicin (EC_50_: 0.37 ± 0.01 μM) and rifabutin (EC_50_: 1.54 ± 1.0 μM) enhanced the reporter signal (*E*_max_) approximately 5-fold compared to untreated cells (Fig. 2). Overexpression of PXR.2 yielded similar activation dynamics, which were comparable and non-significantly different to PXR.1 (rifampicin EC_50_: 0.47 ± 0.05 μM; rifabutin EC_50_: 1.4 ± 0.78 μM; *E*_max_ values approximately 4-fold compared to untreated cells). In contrast, the reporter signals weakly but statistically significantly increased when PXR.3-transfected cells were treated with rifampicin (80% increase in *E*_max_, *P* = 0.0016), while rifabutin had no effect. The poor activation by rifampicin resembled the activation dynamics of reporter assays without PXR overexpression (endogenous PXR only; 90% increase in *E*_max_ compared to untreated control) (Suppl. Fig. S1), suggesting that PXR.3 overexpression did not functionally contribute to the cell’s overall PXR activity.

### *NR1I2* mRNA expression in LS180 cells

**Fig. 3:**
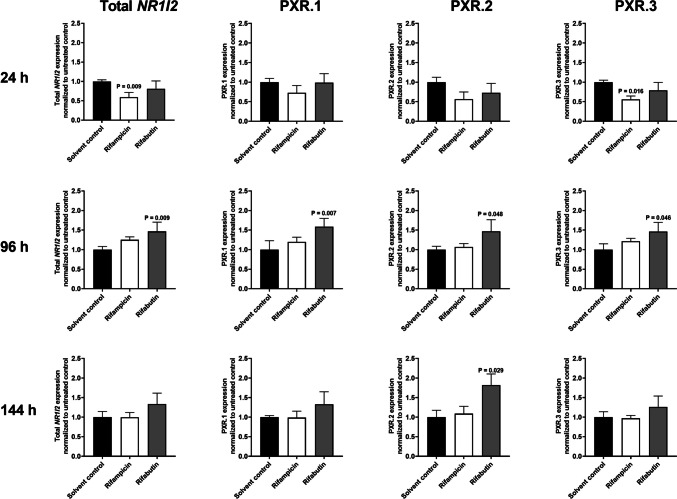
Impact of 10 μM rifampicin (white bars) or rifabutin (gray bars) for 24 h, 96 h, or 144 h on mRNA expression of total *NR1I2* or its splice variants in LS180 cells, normalized to solvent control (black bars). Data shown is the mean ± S.D. of four independent biological replicates. Statistical significance was evaluated by non-parametric Kruskal-Wallis test and Dunn’s test, correcting for multiple comparisons

LS180 cells were exposed for 24, 96, and 144 h to 10 μM of each rifamycin. Total *NR1I2* mRNA expression was reduced by 40% after 24-h treatment with rifampicin (*P* = 0.009), while rifabutin enhanced the expression by 46% after 96-h exposure (*P* = 0.009) (Fig. 3). The mRNA expression of the *NR1I2* splice variant PXR.1 was increased by 58% after 96-h treatment with rifabutin (*P* = 0.007). Likewise, rifabutin enhanced the mRNA expression of PXR.2 by 46% after 96 h (*P* = 0.048) and by 82% after 144 h (*P* = 0.029). PXR.3 mRNA expression was reduced by 45% after 24-h treatment with rifampicin (*P* = 0.016), but was increased by 46% after rifabutin exposure for 96 h (*P* = 0.046).

### *NR1I2* mRNA expression in primary human hepatocytes

**Fig. 4:**
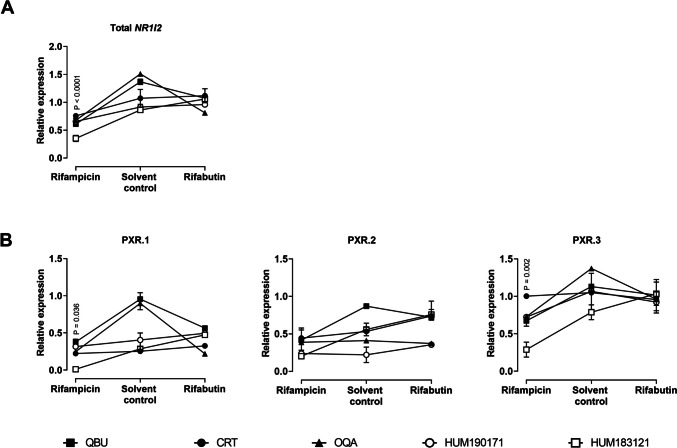
Relative mRNA expression of total *NR1I2* (**A**) or its splice variants (**B**) in primary human hepatocytes after treatment with rifampicin or rifabutin (both 10 μM) for 48 h. Data points shown are the mean ± S.D. of hepatocyte samples (pooled from three independent experiments), evaluated by two technical replicates (PCR runs). Statistical significance was evaluated by non-parametric Kruskal-Wallis test and Dunn’s test (correcting for multiple comparisons), comparing all solvent control hepatocyte samples with all rifampicin-treated or rifabutin-treated hepatocyte samples

*NR1I2* splice variant mRNA expression was also evaluated in primary human hepatocytes from different donors after 48-h treatment with 10 μM rifampicin or rifabutin and compared to solvent controls (Fig. 4). Among the different donors, expression levels differed substantially at baseline (solvent control). Rifampicin lowered the expression of total *NR1I2* (*P* < 0.0001, Fig. 4A), PXR.1 (*P* = 0.036), and PXR.3 (*P* = 0.002), whereas rifabutin had no effects. There was no significant impact on PXR.2 expression after rifamycin treatment (Fig. 4B).

### *NR1I2* mRNA expression in samples of healthy liver and colon tissue

**Fig. 5:**
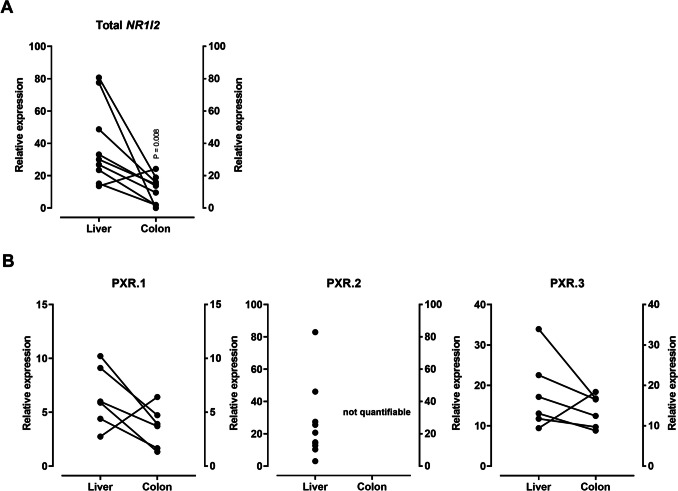
Relative mRNA expression of total *NR1I2* (**A**) or its splice variants (**B**) in matched samples of healthy liver and colon. Data points shown are the mean relative expression of each sample, evaluated by two technical replicates (PCR runs). Statistical significance was evaluated by non-parametric Wilcoxon matched-pairs signed-rank test. Samples with unreliable housekeeping gene expression or outliers identified by ROUT testing (Q-level at 1%) were excluded from the analysis

In 8 of 9 individuals, total *NR1I2* mRNA expression was lower in the colon than in the corresponding hepatic samples of the respective patient (*P* = 0.008) (Fig. 5A). This difference is likely caused by high hepatic PXR.2 expression, whereas the colon expressed PXR.2 below the detection limit. There were no differences in the tissue-specific expression of PXR.1 or PXR.3 mRNA (Fig. 5B). When comparing the mRNA expression levels of the PXR splice forms, PXR.1 mRNA expression levels were the lowest both in the liver (hepatic PXR.1 vs. hepatic PXR.2: *P* = 0.009; hepatic PXR1. vs. hepatic PXR.3: *P* = 0.049) and colon samples (colon PXR.1 vs. colon PXR.3: *P* = 0.031). In addition, hepatic PXR.2 and hepatic PXR.3 mRNA expression levels were similar.

## Discussion

We have recently shown that rifabutin has considerably fewer effects on *CYP3A4* and *ABCB1* than rifampicin, especially when normalized to actual intracellular rifamycin concentrations (Nilles et al. 2023). In order to clarify the possible molecular mechanisms leading to the different inductive effects of rifampicin and rifabutin, we investigated three hypotheses on the pharmacology of PXR:

First, rifampicin might be a more potent activator of certain *NR1I2* splice variants than rifabutin, leading to a higher overall cellular PXR-activating effect and thus greater induction of *CYP3A4* or *ABCB1*. However, selective overexpression of PXR.1, PXR.2, or PXR.3 and subsequent reporter gene assays did not reveal pharmacodynamic differences between the two antibiotics, suggesting that variable activation of PXR splice forms was not the reason of unequal drug disposition gene inductions by these two compounds. Furthermore, the data confirmed that PXR.3 can hardly be activated by rifampicin (Lin et al. 2009; Hustert et al. 2001) and revealed that this is also true for rifabutin. After PXR.3 transfection (= overexpression), firefly luminescence in fact increased upon rifampicin treatment, but this signal likely resulted from the cells’ endogenous functional PXR (e.g., endogenous PXR.1).

The second tested hypothesis was that rifampicin might be the stronger *CYP3A4* or *ABCB1* inducer through concurrently enhancing the transcription and thus the expression of the PXR gene or its activatable splice variants (fast-forward mechanism). Consequently, mRNA expression levels in LS180 cells and primary human hepatocytes were assessed after exposure to 10 μM rifampicin or rifabutin for variable exposure times. Rifampicin however lowered the mRNA expression of total *NR1I2* and PXR.3 in both primary human hepatocytes and LS180 cells, especially after 24 h of treatment, clearly contradicting the hypothesized fast-forward mechanism. This rifampicin-mediated suppression of total *NR1I2* after 24-h drug treatment is consistent with previously reported data (Smutny et al. 2020, Bailey et al. 2011) and seems to be (at least in part) mediated by the PXR.3 suppression because PXR.1 and PXR.2 remained unaffected in LS180 cells. In contrast, rifabutin enhanced the mRNA expression of total *NR1I2* in LS180 cells after 96-h drug treatment, which was likely due to enhancements of all three splice variants evaluated. The rifabutin-mediated enhancement of PXR.3 in LS180 cells might have functional consequences because the protein encoded by PXR.3 can hardly be activated or can even out-balance activation of PXR.1 (Lin et al. 2009). For instance, when cells had been co-transfected with a fixed amount of a PXR.1-encoding plasmid (10 ng) plus variable amounts of a PXR.3-encoding plasmid and subsequently treated with rifampicin, the PXR.1 activation was “dose-dependently” compensated by increasing amounts of PXR.3 plasmids (Lin et al. 2009). Accordingly, our data suggest that by concurrently enhancing PXR.3 expression, rifabutin might weaken its activation of PXR.1, partly explaining rifabutin’s weaker net effect on *CYP3A4* or *ABCB1* in LS180 cells (Nilles et al., 2023). However, this might only be of relevance when *NR1I2* and its splice variants are expressed in vivo at all.

Accordingly, our third hypothesis was that the drug-responsive PXR forms are differentially expressed in the liver, a major site of drug metabolism. Moreover, we additionally evaluated expression levels in the colon, a little-studied site of PXR expression (van de Kerkhof 2008). Our data confirmed that total *NR1I2* expression levels vary considerably among individuals (Gardner-Stephen et al. 2004) and showed that its expression level in the colon is significantly lower than in the liver. This is in line with previous findings on the PXR target genes. For instance, CYP3A4 and P-gp are highly expressed in the liver but only small amounts of the respective mRNA or protein were detected in the colon (Canaparo et al. 2007; Berggren et al. 2007; McKinnon et al. 1995). The PXR splice variants also tended to be less strongly expressed in the colon than in the liver, but the difference was not statistically significant. Together, the liver certainly is a major site of drug disposition gene induction given its high expression of PXR (mainly driven by high expression of activatable PXR.2). In contrast, the colon expresses less functional PXR, making the colon a less likely site of induction-mediated drug-drug interactions (Huppertz et al. 2019).

This study has its strengths and weaknesses. A major limitation is that expression was not studied in small intestinal tissue, which is as important as the liver for induction-related drug-drug interactions for orally administered drugs (Thelen & Dressman 2009; Galetin et al. 2008; Gorski et al. 2003). In addition, we have only assessed mRNA (but no protein) levels of the *NR1I2* splice variants. However, splicing occurs at the level of pre-mRNA and eventually quantifying the mature mRNA forms through PCR thus seems an appropriate approach. Another limitation is the unequal expression of housekeeping genes in the liver and colon samples, so comparative conclusions about the respective expression levels should be taken with caution. The strength of this study is that well-designed, specific primer sequences and their binding sites were used. Previous primer sequences were in fact published (Fukuen et al. 2002; He et al. 2006), but during the establishment of our PCR protocols, these sequences seemed to lead to amplification side products (detected during melting curve analysis), and splice variant sequences were partly overlapped or were not specific. In contrast, the primers indicated here demonstrably led to distinct single PCR products and yielded first information on mRNA expression levels of three major *NR1I2* splice variants in matched samples of healthy liver and colon. Another strength is the use of different cell systems (cell line, primary cells, and liver and colon specimens) which allows the drawing of profound conclusions.

## Conclusion

While rifampicin and rifabutin activated PXR splice variants similarly, rifampicin tended to decrease the expression of *NR1I2* splice variants and rifabutin rather increased them, including the non-activatable PXR.3 splice form. However, these changes appear too small to explain the observed in vivo and in vitro differences in CYP3A4 and P-gp induction by rifampicin or rifabutin.

### Supplementary information

ESM 1 (PNG 56 kb)

High resolution image (TIF 232 kb)

ESM 2 (DOCX 13 kb)

## Data Availability

Data is available upon request.

## Abbreviations

PXR: Pregnane X receptor
CYP3A4: Cytochrome P-450 isozyme 3A4
P-gp: P-Glycoprotein
