# Supplementary figures and images for: The differences in drug disposition gene induction by rifampicin and rifabutin are unlikely due to different effects on important pregnane X receptor (NR1I2) splice variants
Source: Naunyn Schmiedebergs Arch Pharmacol. 2023 Oct 18;397(4):2485–96. doi: 10.1007/s00210-023-02768-z (PMC10933196; doi:10.1007/s00210-023-02768-z)

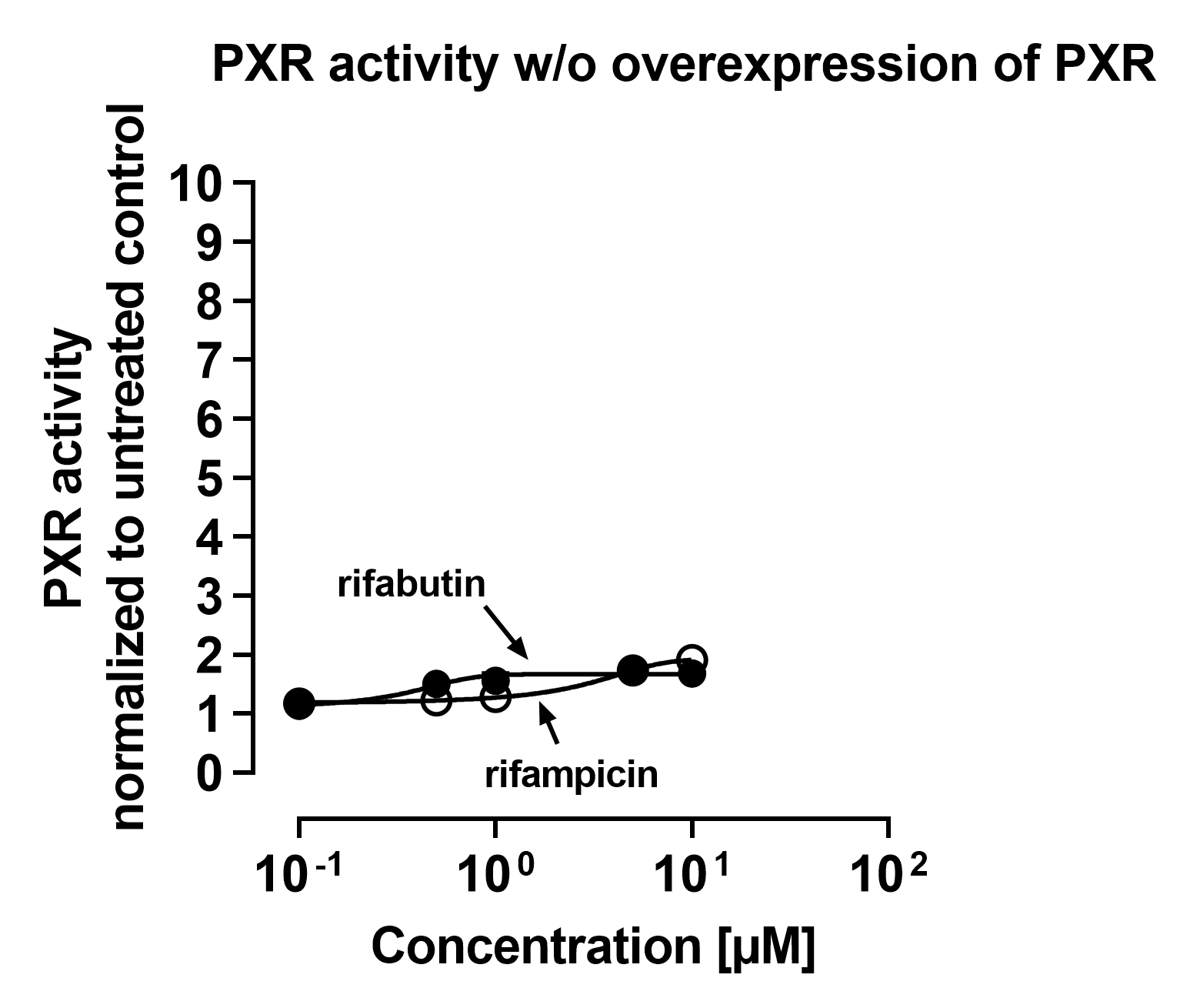

Supplement: Supplementary file 1 — (PNG 56 kb) [file 210_2023_2768_Fig6_ESM.png]

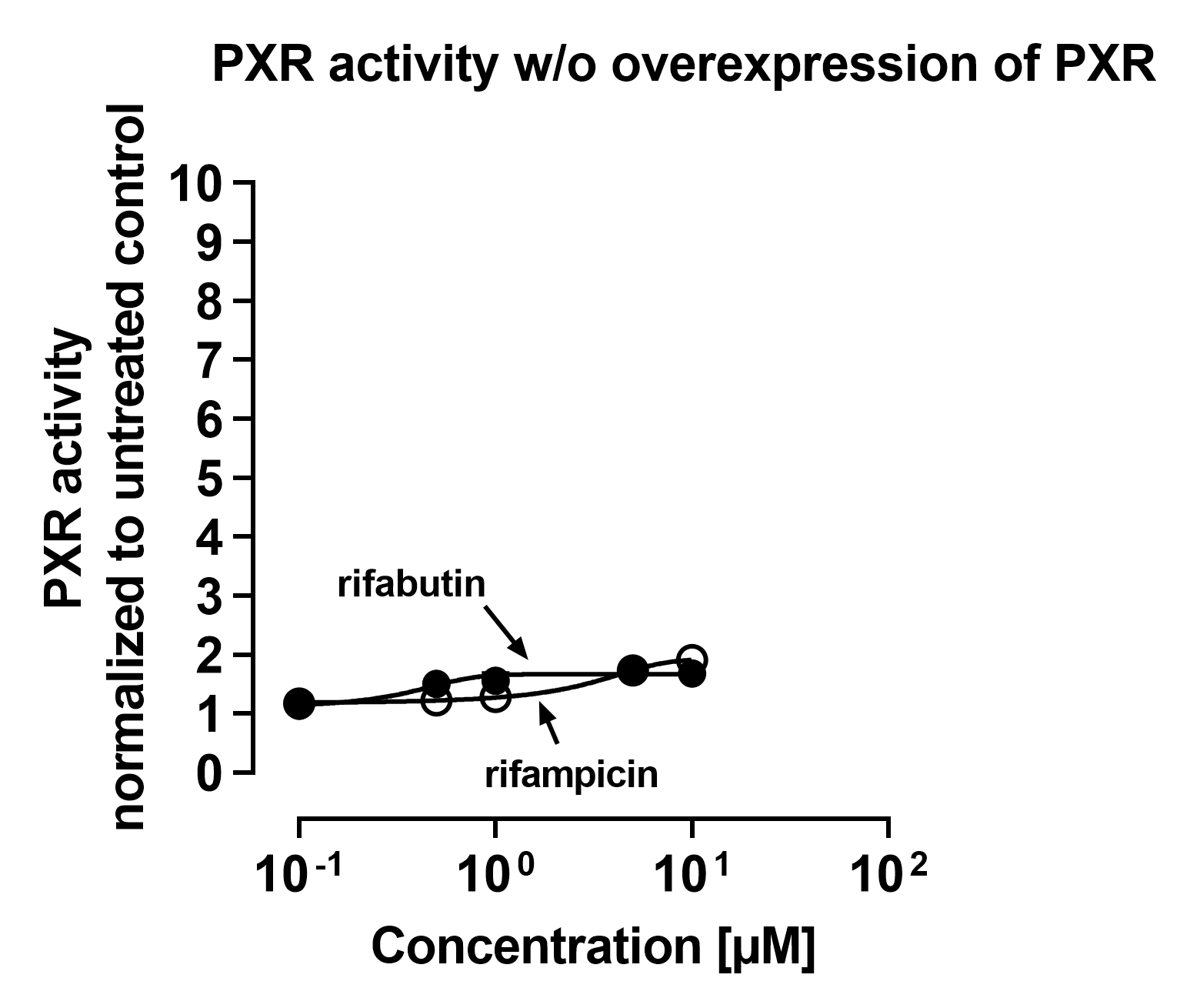

Supplement: Supplementary file 2 — High resolution image (TIF 232 kb) [file 210_2023_2768_MOESM1_ESM.docx]
