# Supplementary material for: The differences in drug disposition gene induction by rifampicin and rifabutin are unlikely due to different effects on important pregnane X receptor (NR1I2) splice variants
Source: Naunyn Schmiedebergs Arch Pharmacol. 2023 Oct 18;397(4):2485–96. doi: 10.1007/s00210-023-02768-z (PMC10933196; doi:10.1007/s00210-023-02768-z)
Supplement: Supplementary file 3 — (DOCX 13 kb) [file 210_2023_2768_MOESM2_ESM.tif]

**The differences in drug disposition gene induction by rifampicin and rifabutin are unlikely due to different effects on important pregnane X receptor (*NR1I2*) splice variants**

Julie Nilles^1,2^, Johanna Weiss^1^, Martin Masin^1^, Christopher Tuffs^3^, Moritz J. Strowitzki^3^, Walter E. Haefeli^1^, Stephanie Ruez^2^, Dirk Theile^1^

1) Department of Clinical Pharmacology and Pharmacoepidemiology, Heidelberg University Hospital, Im Neuenheimer Feld 410, 69120 Heidelberg, Germany

2) Boehringer Ingelheim Pharma GmbH & Co. KG, Birkendorfer Str. 65, 88397 Biberach an der Riss, Germany.

3) Departments of General, Visceral, and Transplantation Surgery, Heidelberg University Hospital, Im Neuenheimer Feld 420, 69120 Heidelberg, Germany

**Supplemental Material**

Fig. S1: Relative PXR activity in LS180 cells after 72 h exposure to rifampicin (open circles) or rifabutin (closed circles) without (w/o) overexpression of PXR. Data shown is the mean ± S.D. of the two to three independent biological replicates with triplicates for each concentration. Data were fitted according to an E_max_ model (four parameter-logistic equation; variable slope).
